# C9orf72 dipeptides disrupt the nucleocytoplasmic transport machinery and cause TDP-43 mislocalisation to the cytoplasm

**DOI:** 10.1038/s41598-022-08724-w

**Published:** 2022-03-21

**Authors:** Sarah Ryan, Sara Rollinson, Eleanor Hobbs, Stuart Pickering-Brown

**Affiliations:** 1 Division of Neuroscience and Experimental Psychology, Faculty of Biology, Medicine and Health, University of Manchester, Oxford Road, Manchester, UK; 2 Geoffrey Jefferson Brain Research Centre, The Manchester Academic Health Science Centre, Northern Care Alliance NHS Group, University of Manchester, Manchester, UK

**Keywords:** Cell biology, Neuroscience, Diseases, Medical research

## Abstract

A repeat expansion in C9orf72 is the major cause of both frontotemporal dementia and amyotrophic lateral sclerosis, accounting for approximately 1 in 12 cases of either disease. The expansion is translated to produce five dipeptide repeat proteins (DPRs) which aggregate in patient brain and are toxic in numerous models, though the mechanisms underlying this toxicity are poorly understood. Recent studies highlight nucleocytoplasmic transport impairments as a potential mechanism underlying neurodegeneration in C9orf72-linked disease, although the contribution of DPRs to this remains unclear. We expressed DPRs in HeLa cells, in the absence of repeat RNA. Crucially, we expressed DPRs at repeat-lengths found in patients (> 1000 units), ensuring our findings were relevant to disease. Immunofluorescence imaging was used to investigate the impact of each DPR on the nucleus, nucleocytoplasmic transport machinery and TDP-43 localisation. DPRs impaired the structural integrity of the nucleus, causing nuclear membrane disruption and misshapen nuclei. Ran and RanGAP, two proteins required for nucleocytoplasmic transport, were also mislocalised in DPR-expressing cells. Furthermore, DPRs triggered mislocalisation of TDP-43 to the cytoplasm, and this occurred in the same cells as Ran and RanGAP mislocalisation, suggesting a potential link between DPRs, nucleocytoplasmic transport impairments and TDP-43 pathology.

## Introduction

Frontotemporal dementia (FTD) and amyotrophic lateral sclerosis (ALS) are two devastating neurodegenerative diseases with considerable clinical, genetic and pathological overlap. A large G_4_C_2_ repeat expansion in C9orf72 is the major known cause of both FTD and ALS (C9FTD/ALS), accounting for approximately 1 in 12 cases^1,2^. The expanded region of C9orf72 is transcribed to produce long, repetitive G_4_C_2_ RNA transcripts which form intraneuronal nuclear foci in patient brain and sequester RNA-binding proteins^1^. The repeat region is also translated via unconventional repeat-associated non-ATG translation to generate five distinct dipeptide repeat proteins (DPRs): glycine–alanine (GA), glycine–arginine (GR), proline–arginine (PR), alanine–proline (AP) and glycine–proline (GP). These peptides form inclusions in patient brain^3,4^ and are highly toxic in various cell culture models^5–8^, as well as in *Drosophila*^9,10^, zebrafish^11^ and mice^12–16^. However, the mechanisms underlying DPR toxicity are unknown.

In addition to DPR pathology, the FTD/ALS-related protein TDP-43 (transactive response DNA-binding protein 43) also aggregates in C9orf72-positive patient brain^1^. TDP-43 is mislocalised from the nucleus to cytoplasm where it becomes abnormally hyperphosphorylated and forms insoluble intraneuronal inclusions. TDP-43 pathology correlates well with neuronal loss in C9FTD/ALS, leading to the proposal of an “amyloid-like cascade” hypothesis of neurodegeneration, whereby the C9orf72 expansion produces RNA foci and DPRs which somehow trigger TDP-43 pathology and this causes neuronal death^17^. However, the link between RNA foci/DPRs and TDP-43 mislocalisation is not well established, and there are likely multiple mechanisms through which products of the C9orf72 expansion impair cellular function which converge to cause neurodegeneration in FTD/ALS.

Recent literature has highlighted defects in nucleocytoplasmic transport as an important feature of C9FTD/ALS, with large-scale genetic screening studies identifying a number of key nucleocytoplasmic transport genes as modifiers of toxicity in fly and yeast models^18–22^. These studies led to the novel hypothesis that the C9orf72 expansion causes neurodegeneration by disrupting nucleocytoplasmic transport, with a wide range of downstream consequences for cellular processes necessary for normal function and survival. However, the exact mechanisms underlying nucleocytoplasmic transport impairments in C9FTD/ALS remain unclear. Much of the literature published to date utilises models which express pure G_4_C_2_ repeat sequences, and therefore all five DPRs as well as G_4_C_2_ repeat RNA will be produced in those models, rendering it impossible to determine the specific cause of any observed phenotypes. Furthermore, those models which use alternative-codon sequences to express DPRs in the absence of G_4_C_2_ repeat RNA do so at extremely short repeat-lengths which are not found in patients. We have previously shown that repeat-length determines the impact of DPRs on cellular function and toxicity, with longer repeats causing more severe phenotypes^8,11^. As such, it is essential to model DPR pathology at repeat-lengths found in patients wherever technically possible, to ensure relevance to human disease.

Here we used alternative-codon sequences for expression of DPRs in the absence of G_4_C_2_ repeat-RNA, to investigate the specific impact of each dipeptide on the nuclear membrane and nucleocytoplasmic transport machinery. We also investigated the link between C9orf72 dipeptides and TDP-43, which is mislocalised to the cytoplasm in the brain of expansion-carrying patients. Crucially, we used previously characterised constructs^8^ to express each DPR at long repeat-lengths which are known to be found in patients (> 1000 units), ensuring our findings are relevant to human disease.

## Results

### DPRs cause structural damage to the nucleus and nuclear membrane

**Figure 1:**
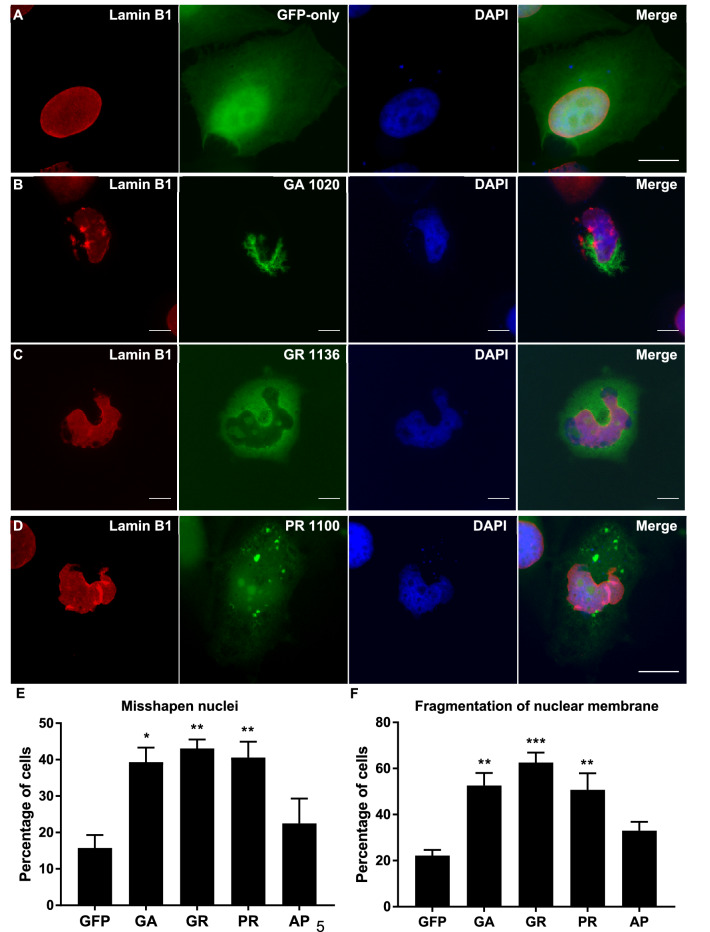
Abnormal nuclei in DPR-expressing cells. (**A**–**D**) Lamin B1 was used as a marker for the nuclear membrane (red). HeLa cells transfected with GFP-tagged DPR constructs or empty pEGFP-N1 vector as a GFP-only control were fixed and stained 48 h post-transfection. Nuclei were frequently “horseshoe-shaped” or excessively folded in cells containing GA_1020_ (*P* = 0.0131), GR_1136_ (*P* = 0.0052) or PR_1100_ inclusions (*P* = 0.0096). AP_1024_ did not cause nuclear abnormalities (data not shown; *P* = 0.6797). In addition, the nuclear membrane is fragmented in cells expressing GA_1020_ (*P* = 0.0047), GR_1136_ (*P* = 0.0006) or PR_1100_ (*P* = 0.0071), but not AP_1024_ (data not shown; *P* = 0. 3865). DAPI was used to stain nuclei (blue). Scale bars indicate 15 μm. (**E**, **F**) Graphs shows percentage of transfected cells with misshapen nuclei (**E**) or fragmented nuclear membranes (**F**). n = 3, with a minimum of 30 cells analysed for each independent replicate. Data was analysed by one-way ANOVA with Dunnett’s multiple comparison test. Error bars indicate SEM.

Immunofluorescence imaging was performed on HeLa cells transfected with GA_1020_, GR_1136_, PR_1100,_ AP_1024_ or empty pEGFP-N1 vector as a GFP-only control. Cells were stained with DAPI to visualise the nucleus and an antibody against lamin-B1, a key component of the lamin matrix which forms the nuclear membrane. A large proportion of cells containing GA, GR or PR inclusions had misshapen nuclei, indicating that DPRs cause structural abnormalities in the nucleus. Rather than forming smooth, rounded structures, nuclei were excessively folded to form “horseshoe” shapes or cause severe creasing in the nuclear membrane (Fig. 1). Quantification of this phenotype showed that DPRs significantly increased the percentage of cells with misshapen nuclei, with 39.3% of cells containing GA inclusions affected (*P *= 0. 0131).

43.1% for GR (*P *= 0.0052), and 40.6% for PR (*P *= 0.0096) compared to 15.7% for GFP-only control (n = 3). Lamin B1 staining of cells containing GA, GR or PR inclusions demonstrated that the lamin matrix had a frayed appearance, suggesting that DPRs caused fragmentation of the nuclear membrane (Fig. 1). Again, quantification showed that all three DPRs significantly increased the percentage of cells with this phenotype; 52.6% of cells containing GA inclusions had fragmented lamin B1 staining (*P *= 0.0047), 62.6% for GR (*P *= 0.0006), and 50.7% for PR (*P *= 0. 0071) compared to 22.2% for GFP-only control (n = 3). AP_1024_ did not cause structural damage to the nucleus, with only 22.5% of AP-transfected cells exhibiting misshapen nuclei (*P *= 0.6797) and 33.0% exhibiting nuclear membrane fragmentation (*P* = 0.3865; images not shown, quantification in Fig. 1E, F, respectively). Since GA, GR and PR all significantly increased the percentage of cells with either misshapen nuclei or fragmentation of the nuclear membrane, Tukey’s multiple comparisons tests were also performed to compare the severity of each phenotype between each DPR treatment (Table 1). No significant differences were observed with the exception of the percentage of cells with nuclear fragmentation caused by GR compared to AP (*P *= 0.0113).

**Table 1:** Since the statistical analysis in Fig. 1 showed that several DPRs significantly increased the frequency of nuclear abnormalities but to different extents, Tukey’s multiple comparisons tests were also performed to compare between each DPR treatment.

Misshapen nuclei			Fragmentation of nuclear membrane	
DPRs	*P* value	Significance	*P* value	Significance
GA vs. GR	0.9735	NS	0.6191	NS
GA vs. PR	0.9996	NS	0.9985	NS
GA vs. AP	0.1327	NS	0.1031	NS
GR vs. PR	0.9940	NS	0.4674	NS
GR vs. AP	0.0538	NS	0.0113	*
PR vs. AP	0.0989	NS	0.1557	NS

*NS* not significant.

### Components of the Ran cycle are mislocalised in DPR-transfected cells

**Figure 2:**
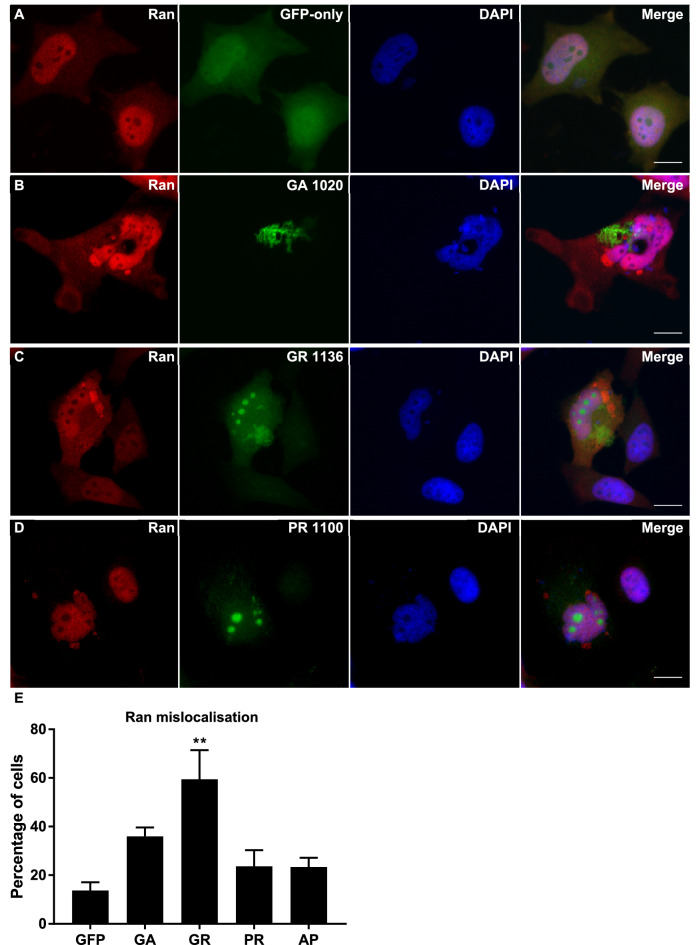
Ran was mislocalised to the cytoplasm in cells expressing DPRs. (**A**–**C**) HeLa cells transfected with GFP-tagged DPR constructs or empty pEGFP-N1 vector as a GFP-only control were fixed and stained 48 h post-transfection. Ran (red) is a predominately nuclear protein in GFP-only control cells (**A**) but frequently accumulated in the perinuclear region in cells expressing GA_1020_ (**B**), GR_1136_ (**C**) or PR_1100_ (**D**). The percentage of cells with Ran mislocalisation was increased by expression of both GA (*P* = 0.1226) and PR (*P* = 0.0025), however this was only statistically significant for GR Scale bars indicate 15 μm. (**E**) Quantification of Ran staining intensity in the nucleus compared to the perinuclear region. Ran was considered to be mislocalised if less than 90% of staining was nuclear. n = 3, with a minimum of 30 cells analysed for each independent replicate. Data was analysed by one-way ANOVA with Dunnett’s multiple comparison test. Error bars indicate SEM.

Next, we investigated the impact of DPR expression on the nucleocytoplasmic transport machinery. The Ran cycle is an important cellular process that provides the energy to actively transport proteins across the nuclear membrane. Immunofluorescence staining was performed for Ran and RanGAP, two key components of the Ran cycle, in HeLa cells transfected with DPR constructs or GFP-only control vector. In control cells, Ran was a predominantly nuclear protein, with some diffuse expression in the cytoplasm (Fig. 2A). However, in many cells containing GR inclusions, Ran mislocalised to the cytoplasm where it accumulated close to the nuclear membrane (Fig. 2C/E). The intensity of Ran staining was measured in the nucleus compared to the perinuclear region, and >90% of Ran staining was contained within the nucleus in the vast majority (86.3%) of GFP-only control cells. 90% of staining within the nucleus was therefore set as a threshold for “normal” Ran localisation. In GR-expressing cells, the percentage of cells with abnormal distribution of Ran was significantly increased from 13.7 to 59.5% (*P *= 0.0025), indicating that GR caused mislocalisation of Ran to the cytoplasm. Accumulation of Ran in the cytoplasm was also observed in cells containing GA inclusions, however this occurred less frequently (36.0% of cells) and was not statistically significant (*P *= 0.1226; Fig. 2B/E). Neither PR (Fig. 2D) nor AP caused mislocalisation of Ran (*P *= 0.6928 and 0.7109 respectively; Fig. 2E), demonstrating that DPRs may differentially impact the nucleocytoplasmic transport machinery.

**Figure 3:**
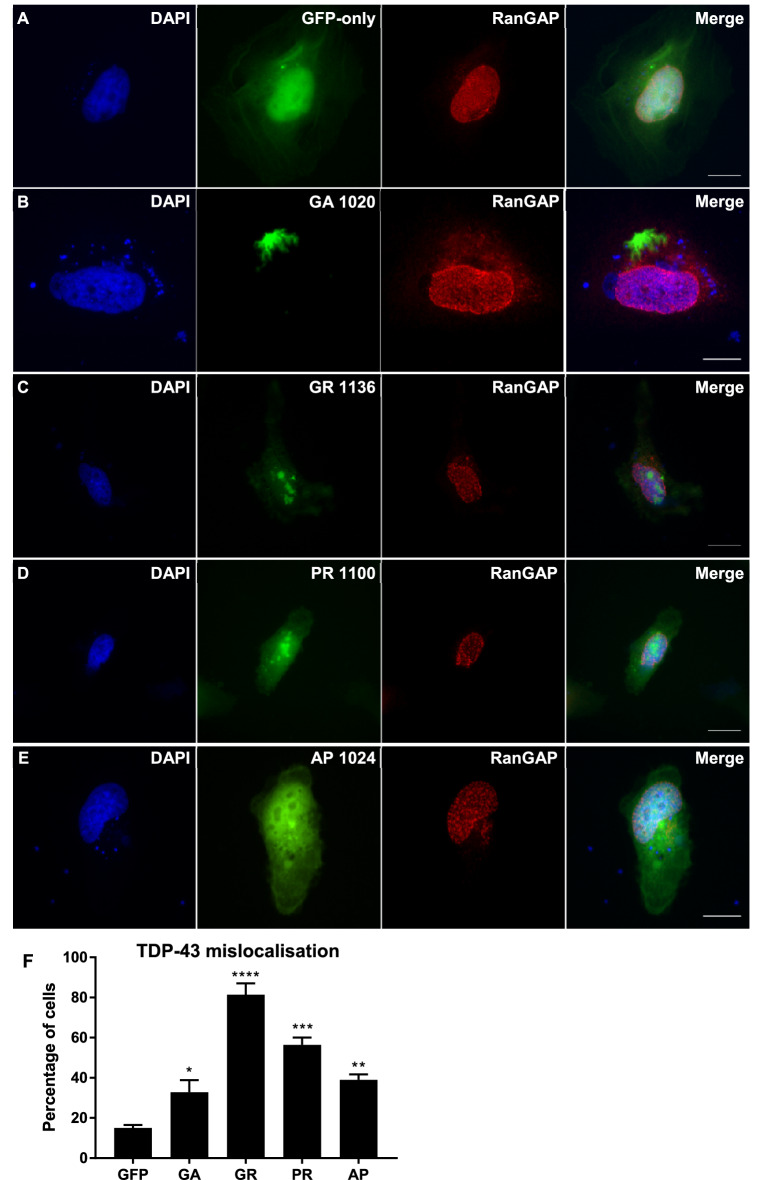
RanGAP formed granular inclusions in cells expressing DPRs. (**A**–**E**) HeLa cells transfected with GFP-tagged DPR constructs or empty pEGFP-N1 vector as a GFP-only control were fixed and stained 48 h post-transfection. RanGAP (red) is diffusely expressed in the nucleus of GFP-only control cells (**A**) but formed granular inclusions in the nuclei of a proportion of cells expressing DPRs (**B**–**E**). Scale bars indicate 15 μm. (**F**) Graph shows the percentage of cells exhibiting granular RanGAP inclusions, which was significantly increased by expression of GA_1020_ (*P *= 0.0483)**,** GR_1136_ (*P* < 0.0001), PR_1100_ (*P* = 0.0002) or AP_1024_ (*P* = 0.0096). n = 3, with a minimum of 30 cells analysed for each independent replicate. Data was analysed by one-way ANOVA with Dunnett’s multiple comparison test. Error bars indicate SEM.

RanGAP was also predominantly a nuclear protein, with relatively diffuse nuclear staining observed in GFP-only control cells. DPR expression caused RanGAP to form punctate, granular accumulations (Fig. 3). Interestingly, all DPRs tested significantly increased the percentage of cells with abnormal accumulation of RanGAP, (GA 32.8%, *P *= 0.0483; GR 81.4%, *P *< 0.0001; PR 56.4%, *P *= 0.0002; AP 38.9%, *P *= 0.0096 compared to GFP-only control of 15.0%), although the severity of this phenotype varied between peptides with arginine-rich dipeptides causing RanGAP accumulation most frequently. The severity of RanGAP mislocalisation was also significantly different between most of the DPR treatments (Table 2). These findings demonstrate that DPRs differentially cause mislocalisation of two key components of the Ran cycle, most likely contributing to the nucleocytoplasmic transport impairments reported to occur in C9FTD/ALS.

**Table 2:** Since the statistical analysis in Fig. 3 showed that all DPRs significantly increased the frequency of RanGAP mislocalisation but to different extents, a Tukey’s multiple comparisons test was also performed to compare between each DPR treatment.

DPRs	*P* value	Significance
GA versus GR	< 0.0001	****
GA versus PR	0.0210	*
GA versus AP	0.8496	NS
GR versus PR	0.0145	*
GR versus AP	0.0003	***
PR versus AP	0.0983	NS

*NS* not significant.

### DPRs cause mislocalisation of TDP-43 to the cytoplasm

**Figure 4:**
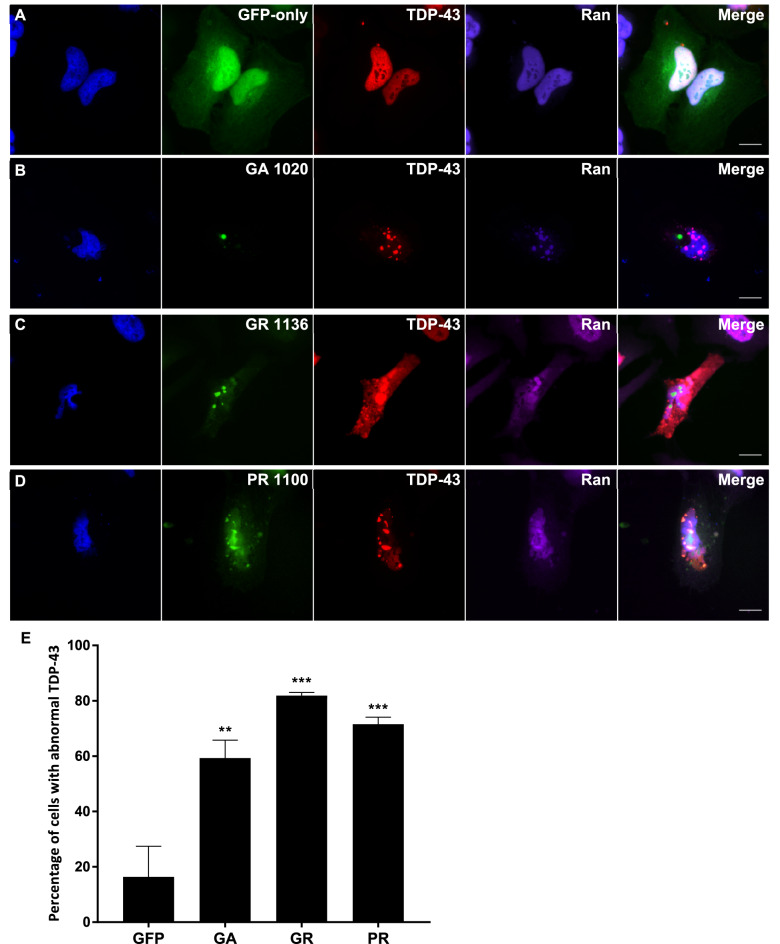
DPRs caused TDP-43 mislocalisation to the cytoplasm. (**A**–**D**) HeLa cells co-transfected with mCherry-tagged human TDP-43 (red) and GFP-tagged DPR constructs or empty pEGFP-N1 vector as a GFP-only control were fixed and stained 48h post-transfection. TDP-43 was frequently mislocalised to the cytoplasm in cells containing GA, GR or PR inclusions. Many DPR-expressing cells also exhibited some TDP-43 aggregation. Scale bars indicate 15 μm. (**E**) Graph shows the mean percentage of total TDP-43 fluorescence signal which was localised to the cytoplasm. The proportion of TDP-43 in the cytoplasm was significantly increased by expression of GA_1020_ (*P* = 0.0003), GR_1136_ (*P *< 0.0011), and PR_1100_ (*P* = 0.0349) but not AP_1024_ (*P* = 0.9999). n = 3, with a minimum of 30 cells analysed for each independent replicate. Data was analysed by one-way ANOVA with Dunnett’s multiple comparison test. Error bars indicate SEM.

Since DPR expression caused both structural damage to the nucleus and nuclear membrane and disruption to key components of the system that powers nucleocytoplasmic transport, we next investigated whether TDP-43, a nuclear protein known to be mislocalised to the cytoplasm in C9FTD/ALS patient brain, was affected by DPR expression. HeLa cells were co-transfected with an expression construct for mCherry-tagged human TDP-43 and GFP-tagged DPRs or empty pEGFP-N1 control. Immunofluorescence imaging was used to determine whether DPR expression affected the nuclear localisation of TDP-43. Figure 4 shows that GA, GR and PR all caused mislocalisation of TDP-43 to the cytoplasm. In some cells, TDP-43 also formed several small inclusions. Quantification of staining intensity showed that 18.3% of TDP-43 was localised to the cytoplasm in GFP-only control cells, whereas GA, GR and PR significantly increased this to 41.0% (*P *= 0.0003), 37.6% (*P *= 0.0011) and 29.5% (*P *= 0.0349) respectively. AP did not cause TDP-43 mislocalisation, with only 18.7% of staining contained within the cytoplasm (*P *= 0.999; data not shown). Statistical comparisons between each DPR treatment are shown in Table 3.

**Table 3:** Since the statistical analysis in Fig. 4 showed that all DPRs significantly increased the frequency of TDP-43 mislocalisation but to different extents, a Tukey’s multiple comparisons test was also performed to compare between each DPR treatment.

DPRs	*P* value	Significance
GA versus GR	0.9721	NS
GA versus PR	0.2990	NS
GA versus AP	0.0139	*
GR versus PR	0.5953	NS
GR versus AP	0.0349	*
PR versus AP	0.3140	NS

*NS* not significant.

### DPR-induced mislocalisation of TDP-43 and disruption to the Ran cycle occur together

**Figure 5:**
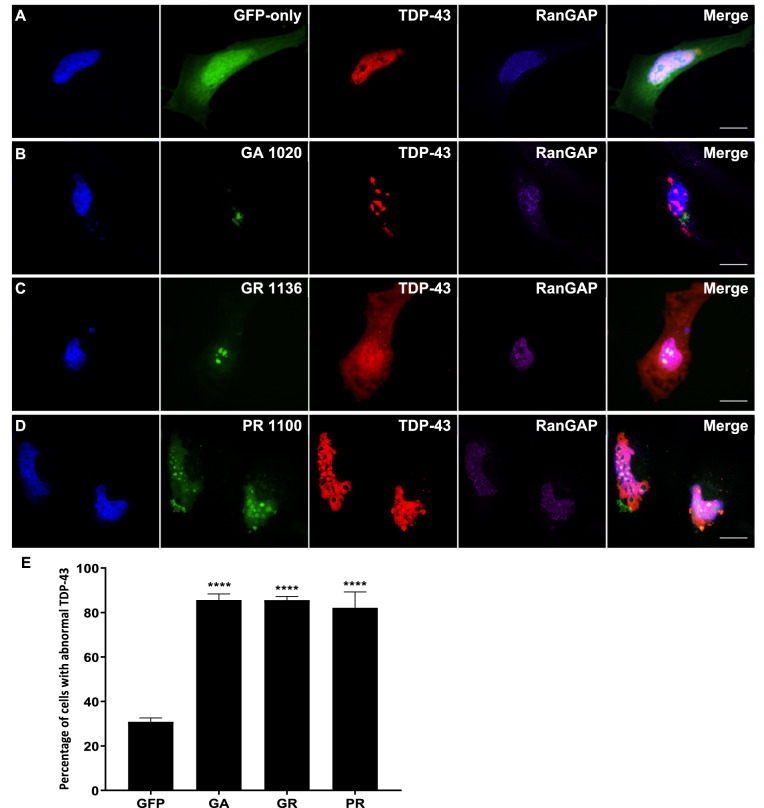
DPR-induced mislocalisation of TDP-43 and Ran occur together. (**A**–**D**) HeLa cells co-transfected with mCherry-tagged human TDP-43 (red) and GFP-tagged DPR constructs or empty pEGFP-N1 vector as a GFP-only control were fixed and stained 48 h post-transfection. Ran was stained using a Cy5-conjugated antibody (purple). Cells exhibiting DPR-induced TDP-43 mislocalisation typically also exhibited mislocalisation of Ran to the cytoplasm. Scale bars indicate 15 μm. (**E**) The mean percentage of cells with abnormal TDP-43 that also exhibited abnormal Ran localisation was significantly increased by GA_1020_ (*P* = 0.0044)**,** GR_1136_ (*P* < 0.0003), and PR_1100_ (*P* = 0.0009). n = 3, with a minimum of 30 cells analysed for each independent replicate. Data was analysed by one-way ANOVA with Dunnett’s multiple comparison test. Error bars indicate SEM.

**Figure 6:**
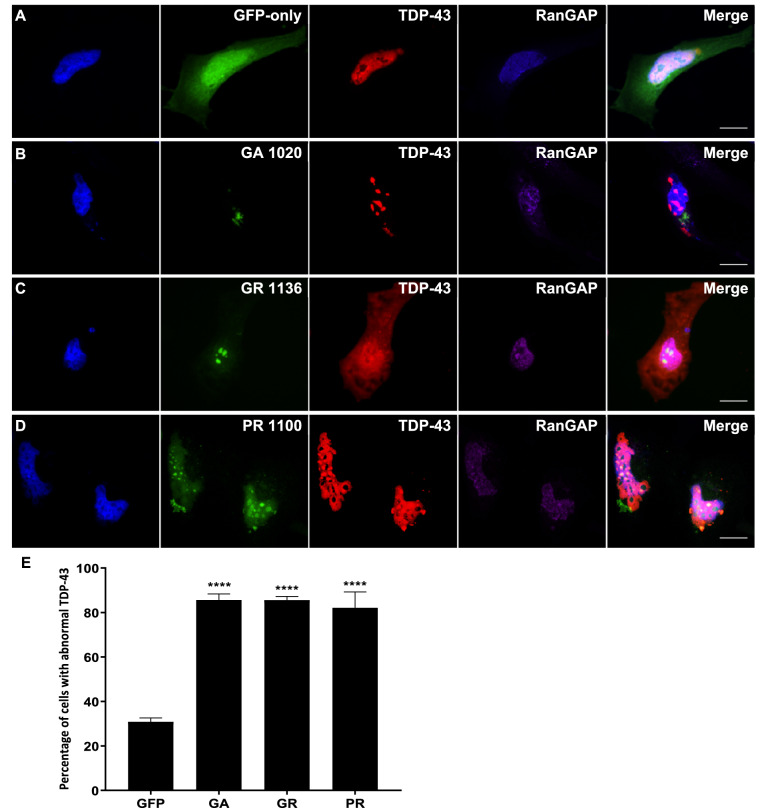
DPR-induced mislocalisation of TDP-43 and RanGAP occur together. (**A**–**D**) HeLa cells co-transfected with mCherry-tagged human TDP-43 (red) and GFP-tagged DPR constructs or empty pEGFP-N1 vector as a GFP-only control were fixed and stained 48 h post-transfection. RanGAP was stained using a Cy5-conjugated antibody (purple). Cells exhibiting DPR-induced TDP-43 mislocalisation typically also exhibited granular accumulation of RanGAP in the nucleus. Scale bars indicate 15 μm. (**E**) The mean percentage of cells with abnormal TDP-43 that also exhibited granular accumulation of RanGAP in the nucleus was significantly increased by GA_1020_ (*P* < 0.0001)**,** GR_1136_ (*P* < 0.0001), and PR_1100_ (*P* < 0.0001). n = 3, with a minimum of 30 cells analysed for each independent replicate. Data was analysed by one-way ANOVA with Dunnett’s multiple comparison test. Error bars indicate SEM.

In order to determine whether the mislocalisation of TDP-43 observed could be linked to DPR-induced disruption to the nucleocytoplasmic transport machinery, we next investigated whether these two things occurred together. Since not all cells containing DPR inclusions exhibited mislocalisation of either Ran, RanGAP or TDP-43, immunofluorescence imaging was performed to determine whether these phenotypes occurred in the same cells. HeLa cells were co-transfected with the mCherry-TDP-43 and DPR-GFP constructs, and a Cy5-conjugated antibody was used to visualise either Ran or RanGAP in these cells (Figs. 5, 6, respectively). The relative intensity of TDP-43 staining in the nucleus and cytoplasm was calculated as described above. Since an average of 18.3% of the TDP-43 staining was previously shown to be contained to the cytoplasm in GFP-only control cells, this was considered the threshold for normal TDP-43 staining, with values above this considered as TDP-43 mislocalisation. The intensity of Ran staining in the nucleus compared to perinuclear area was quantified in cells exhibiting TDP-43 mislocalisation to the cytoplasm, and <90% of Ran staining within the nucleus was considered abnormal as described above. The majority of cells exhibiting DPR-induced TDP-43 mislocalisation also had abnormal mislocalisation of Ran to the cytoplasm (GA 59.3%, *P *= 0.0044; GR 81.9%, *P = *0.0003; PR 71.5%, *P *= 0.0009), indicating that these two phenotypes occur alongside each other. The vast majority of cells exhibiting TDP-43 mislocalisation also exhibited granular RanGAP staining: 85.6% of cells for GA (*P *< 0.0001), GR 85.5% (*P *< 0.0001) and PR 82.1% (*P *< 0.0001). Therefore, both components of the Ran cycle were mislocalised in cells with DPR-induced TDP-43 mislocalisation to the cytoplasm, highlighting the possibility that defects in the nucleocytoplasmic machinery could be the connection between DPRs and TDP-43 pathology.

## Discussion

We have shown that DPRs cause structural damage to the nucleus and fragmentation of the nuclear membrane when expressed at disease-relevant repeat-lengths of over 1000 units. Nuclear membrane fragmentation was previously reported in two *Drosophila* models of C9FTD/ALS, however these models expressed pure G_4_C_2_ repeats (at the very short repeat-lengths of 30 and 58 units), and therefore it was not possible to determine whether this phenotype was caused by DPRs or repeat RNA^20,23^. Here, we demonstrate that breakdown of the nuclear membrane is caused by DPRs, specifically GA, GR and PR but not AP. Our finding is supported by a recent report that staining for both lamin-B1 and lamin-A/C was fragmented in a transgenic mouse expressing PR_50_^13^. Destabilisation of the lamin matrix which forms the nuclear membrane is likely to have detrimental consequences for cellular function and survival in a number of ways. Firstly, impaired structural integrity of the membrane is likely to impact the transport of proteins and other cargoes across it, leading to nucleocytoplasmic transport defects and perhaps breakdown of the barrier which maintains separation of the nuclear and cytoplasmic compartments. Secondly, lamins have several crucial functions besides supporting the structural integrity of the membrane, including roles in DNA repair, chromatin remodelling, transcription and cell migration^24,25^. Furthermore, loss of function mutations in lamins are known to trigger ER stress and activate the unfolded protein response, leading to premature apoptosis^26,27^. It is therefore possible that DPR-induced mislocalisation of lamins directly contributes to neurodegeneration in C9FTD/ALS.

We have also shown that DPRs differently impact the localisation of two proteins required for the Ran cycle: the small GTPase, Ran, and its activator protein, RanGAP. The Ran cycle is an essential process which provides the energy required for active transport of proteins across the nuclear membrane via hydrolysis of RanGTP to RanGDP, creating a concentration gradient across the nuclear membrane^28^. Ran and RanGAP mislocalisation have been previously reported to occur in (G_4_C_2_)_30_ flies and human patient iPSC-derived neurons^20^, however, as with the nuclear membrane fragmentation study, it was not possible to determine the specific cause of this phenotype since these models express G_4_C_2_ repeat RNA as well as all five DPRs_._ We have shown that both phenotypes were caused by DPRs, with all DPRs causing RanGAP to accumulate in the nucleus and primarily GR causing Ran mislocalisation to the cytoplasm. RanGAP puncta have been previously observed in brain tissue from mice expressing GA_50_, supporting our findings^14^. Of note, punctate accumulations of RanGAP are also present in post-mortem brain tissue from C9FTD/ALS patients^20^, demonstrating the relevance of our findings to human disease. Mislocalisation of these key proteins is likely to cause a loss of function phenotype, resulting in nucleocytoplasmic transport impairments. This is supported by genetic screening studies in *Drosophila* expressing (G_4_C_2_)_30_ which found that overexpression of RanGAP rescued toxicity in this model, while RanGAP knockdown worsened the phenotype^20^. Deletion of Ran or RanGAP also increased toxicity in separate fly models expressing (G_4_C_2_)_58_^23^ and PR_50_^21^, highlighting the importance of the Ran cycle in cell survival. Combined with our finding that DPRs impair the structural integrity of the nuclear membrane, our data suggests that DPRs are the driving factor causing nucleocytoplasmic transport defects in C9FTD/ALS. Of particular note, the different DPRs had distinct effects on the nucleocytoplasmic machinery, despite their previously demonstrated toxicity in cell culture models (most potently GA, GR and PR, but also AP when expressed at long repeat-lengths)^5–8^. This demonstrates that the observed phenotypes were not a by-product of DPR toxicity causing cell death through some other mechanism. Functional assays to investigate of the impact of DPR-induced Ran cycle defects on nucleocytoplasmic transport would be beneficial to fully understand their contribution to disease.

Since the nucleocytoplasmic transport machinery was impacted by DPR expression in our model, we next investigated potential downstream consequences of this by assessing the localisation of TDP-43. TDP-43 is a predominantly nuclear RNA-binding protein which is mislocalised to the cytoplasm in C9FTD/ALS patient brain, where it aggregates to form insoluble inclusions. We found that expression of GA, GR and PR, but not AP, all caused TDP-43 to be mislocalised to the cytoplasm, demonstrating a functional link between these disease proteins. This is supported by a recent report of TDP-43 mislocalisation to the cytoplasm in *Drosophila* expressing either pure G_4_C_2_ repeats or alternative-codon DPR sequences encoding GA_64_, GR_64_ or PR_64_^29^. GR_200_ has also recently been found to cause mislocalisation of TDP-43 in mice expressing GR_200_^30^, further supporting our findings. GA_100_ also caused cytoplasmic aggregation of phosphorylated TDP-43 in human neuroblastoma cells^31^. Interestingly, this study found that neither GR_100_ nor PR_100_ caused mislocalisation of TDP-43, indicating that longer repeats may be required to cause a phenotype in this *in vitro* model. Indeed, the authors note that GA triggered the formation of TDP-43 inclusions in a length-dependent manner, and we have previously shown that DPR repeat-length is strongly correlated with toxicity in human cell culture and zebrafish^8,11^.

We found that DPR-induced TDP-43 mislocalisation occurred alongside DPR-induced Ran and RanGAP mislocalisation, implying that dysfunction of the nucleocytoplasmic transport machinery and TDP-43 pathology could be linked in DPR-expressing cells, although further work would be required to definitively confirm this. Ran and TDP-43 mislocalisation also occurred together in the (G_4_C_2_)_30_ fly model and in iPSC-derived neurons from expansion-carrying patients^20^, and our findings suggest that this was most likely triggered by DPRs. Nuclear depletion of Ran has also been reported to occur alongside TDP-43 mislocalisation to the cytoplasm in a GRN^−/−^ mouse model of non-C9FTD, preceding neuronal loss in the retina^32^. Impaired nucleocytoplasmic transport may therefore be a common mechanism leading to TDP-43 mislocalisation and neurodegeneration in different subtypes of FTD.

Our findings show that expression of DPRs in the absence of G_4_C_2_ repeat RNA compromises the structural integrity of the nucleus and nuclear membrane, as well as mislocalising key components of the nucleocytoplasmic transport machinery. We also highlight a potential link between DPRs and TDP-43 pathology, which is supported by recent literature from a *Drosophila* model. It is possible that DPRs impair nucleocytoplasmic transport by disrupting the nuclear membrane and Ran cycle, leading to TDP-43 mislocalisation to the cytoplasm. However, this remains speculative and further work is needed to fully understand the links between DPR-induced mislocalisation of nucleocytoplasmic transport proteins and TDP-43.

## Materials and methods

### Constructs

We previously generated and characterised expression of DPR constructs for expression of GA_1020_, GR_1136_, PR_1100_ and AP_1024_ under the CMV promoter and with a C-terminal GFP-tag, using the pEGFP-N1 vector^8^. To overcome the potential issue of repeat-length instability between preparations of plasmid, each individual tube of DPR construct was size-screened before use by restriction digest and agarose gel as described previously, to ensure repeat-length was correct^8^. Empty pEGFP-N1 vector was used throughout as a GFP-only control. A full length TDP-43 clone (a gift from Professor Leonard Petrucelli, Mayo Clinic, Florida, USA) was used as a template for PCR using the forward primer (5′ GCTCAAGCTTATATGTCTGAATATATTCGGGTA 3′) and reverse (5′ GAGCGGATCCCTACATTCCCCAGCCAGAAGACT 3′) containing *Hind*III and *Bam*HI sites respectively. After digestion the product was ligated in pmCherry-C1 (Clontech) in the same restriction sites to create the pmCherry-C1 TDP-43 WT construct. All constructs were verified by DNA sequencing.

### Cell culture and transfection

HeLa cells (ECACC #93021013) were maintained at 37 °C with 5% CO_2_ in Dulbecco’s Modified Medium (Sigma) supplemented with 10% foetal calf serum (Gibco), 2 mM l-glutamine (Sigma), 100 U/ml penicillin and 100 µg/ml streptomycin (Sigma). Cells were seeded on HCl-treated glass coverslips in 6-well plates overnight and transfected with DPR constructs or empty pEGFP-N1 control vector (800 ng/well) using FuGene HD (Promega; 7.2 µl/well). Cells were incubated at 37 °C for 48 h with a media change performed at 4 h to reduce DPR toxicity. Co-transfection of DPR and TDP-43 vectors was performed by addition of 500 ng of DPR vector or empty pEGFP-N1 control and 500 ng of TDP-43 vector or empty mCherry control and 9 µl FuGene HD. Cells were incubated at 37 °C for 48 h with a media change 4 h post-transfection.

### Immunofluorescence imaging

Cells were fixed in 4% paraformaldehyde 48 h post-transfection and permeabilised with 0.05% Triton-X. Primary antibodies were diluted in phosphate buffered saline (PBS) as follows: anti-lamin B1 (Abcam) 1:500, anti-Ran (Abcam) 1:200, anti-RanGAP (Abcam) 1:100. Donkey anti-rabbit AlexaFluor-594, goat anti-mouse AlexaFluor-594 and goat anti-rabbit Cy5 secondary antibodies were used (Life technologies; 1 drop in 500 µl PBS or 1:500). All antibody incubations were performed at room temperature for 30 min with 3 PBS washes in between. Coverslips were rinsed in MilliQ water and dried overnight before mounting on glass slides using ProLong Diamond Antifade Mounting Media with DAPI (ThermoFisher). All immunofluorescence was performed in triplicate from separate passages of cells.

Images were collected on a Zeiss Axioimager.D2 upright microscope using a 63x/1.4 EC Plan*-*Apochromat objective and captured using a Coolsnap HQ2 camera (Photometrics) through Micromanager software v1.4.23. Specific band pass filter sets for DAPI, FITC, Texas red and Cy5 were used to prevent bleed through from one channel to the next. Images were processed and analysed using Fiji ImageJ (http://imagej.net/Fiji/Downloads). The relative intensity of antibody staining in the nucleus and cytoplasm was determined by calculating corrected total cell fluorescence (CTCF) values on ImageJ. DAPI staining was used to determine the boundaries of the nucleus.

### Statistical analysis

All experiments were performed in triplicate, with a minimum of 30 cells (chosen from random fields of view based on the presence of GFP-tagged DPR expression) analysed per independent replicate. One-way ANOVAs with Dunnett’s test for multiple comparisons were performed for all experiments using GraphPad Prism. *P* values below 0.05 were considered statistically significant.

## Data availability statement

All data generated or analysed during this study are included in this published article.

## Abbreviations

ALS: Amyotrophic lateral sclerosis
AP: Alanine–proline
C9FTD/ALS: C9orf72-linked frontotemporal dementia or amyotrophic lateral sclerosis
CTCF: Corrected total cell fluorescence
DPR: Dipeptide repeat protein
FTD: Frontotemporal dementia
GA: Glycine–alanine
GR: Glycine–arginine
GP: Glycine–proline
PBS: Phosphate buffered saline
PR: Proline–arginine
TDP-43: Transactive response DNA-binding protein 43
